# Characteristics of Selected Adipokines in Ascites and Blood of Ovarian Cancer Patients

**DOI:** 10.3390/cancers13184702

**Published:** 2021-09-20

**Authors:** Marcin Wróblewski, Karolina Szewczyk-Golec, Iga Hołyńska-Iwan, Joanna Wróblewska, Alina Woźniak

**Affiliations:** 1 Department of Medical Biology and Biochemistry, Faculty of Medicine, Collegium Medicum of Nicolaus Copernicus University, ul. Karłowicza 24, 85-092 Bydgoszcz, Poland; karosz@cm.umk.pl (K.S.-G.); joanna.wroblewska@cm.umk.pl (J.W.); al1103@cm.umk.pl (A.W.); 2 Laboratory of Electrophysiology of Epithelial Tissue and Skin, Department of Pathobiochemistry and Clinical Chemistry, Faculty of Pharmacy, Collegium Medicum of Nicolaus Copernicus University, ul. M. Curie Skłodowskiej 9, 85-094 Bydgoszcz, Poland; igaholynska@cm.umk.pl

**Keywords:** adiponectin, ascites, interleukin, MCP-1, ovarian cancer, TIMP-1

## Abstract

**Simple Summary:**

Ovarian cancer is at the forefront of all cancers worldwide. A specific microenvironment for the secretion of various proteins, including adipokines, is formed around the neoplastic tissue. Five of these proteins, namely adiponectin, interleukin 6 (IL-6), interleukin 8 (IL-8), monocyte chemotactic protein-1 (MCP-1) and tissue inhibitor of metalloproteinase-1 (TIMP-1), were found to have a particular effect on metastasis. The review collects data available in the literature on the function and occurrence of these cytokines in ovarian cancer. The collected information will allow for the observation of these proteins in the area of diagnostics and the planning of further scientific research, and will enable the use of the cytokine level determination as biomarkers of neoplastic disease, its progression and prognosis.

**Abstract:**

Ovarian cancer is one of the most common malignancies among women worldwide. The course of the disease is often latent and asymptomatic in the early stages, but as it develops, metastasis occurs, accompanied by accumulation of ascites in the peritoneal cavity. The ascites fluid constitutes a specific microenvironment influencing the processes of carcinogenesis. In ascites, signaling is mediated by various cytokines that control tumor cell proliferation, progression, metastasis, and chemoresistance. Adipokines, secreted into ascites and also appearing in blood, may be markers of ongoing processes related to the development of neoplastic disease. Moreover, a significant influence of adipocyte lipids on the growth of tumors, for which they are one of energy sources, is observed. Adiponectin, interleukin 6 (IL-6), interleukin 8 (IL-8), monocyte chemotactic protein-1 (MCP-1) and tissue inhibitor of metalloproteinase-1 (TIMP-1), discussed in the present review, were found to mediate the effects of omentum metastasis through homing, migration and invasion of ovarian cancer cells. Further research on those adipokines seem to be a natural consequence, allowing for a better understanding of the mechanisms of neoplastic disease and determination of the treatment procedure.

## 1. Introduction

Malignant tumors are one of the most common causes of mortality in the world. In 2018 alone, 295,414 new cases and 184,799 fatal cases of ovarian cancer were reported worldwide, representing 1.6% and 1.9% of all cancers, respectively [1]. Ovarian cancer is the fifth malignancy in terms of mortality in Europe, according to the mortality rate for all malignant tumors, immediately after lung, breast, colorectal and pancreatic cancers [2]. The survival rate for women with diagnosed ovarian cancer has been shown to have decreased in the first decade of the 21st century [2]. Therefore, new methods of effective treatment and early diagnostics should be sought. The onset and course of this disease is silent; at later stages, fluid often collects in the peritoneal cavity. Thus, ascites formed is associated with poor prognosis and deterioration of quality of life and, moreover, constitutes a microenvironment promoting cancer development [2]. Therefore, it seems appropriate to attempt to better understand this microenvironment of free peritoneal fluid in order to elucidate the pathoetiology of ovarian cancer. Such knowledge should improve adaptation and individualization of cancer therapy.

According to histological classification, 90% of all ovarian tumors originate in ovarian epithelium, and the remaining 10% are non-epithelial tumors [3]. Non-epithelial tumors may be derived from reproductive cells. These include, but are not limited to, germinoma, yolk sac carcinoma, teratoma, embryonal carcinoma and embryonal choriocarcinoma. They can also be formed from the sex cords, with examples including folliculoma, thecoma and Sertoli–Leydig cell tumor [3]. Shih and Kurman [4] divided ovarian tumors into two groups. Group I includes slowly growing tumors limited to ovaries, resistant to chemotherapy, but sensitive to hormonal therapy. Group II includes rapidly growing tumors, often spreading parenterally, susceptible to chemotherapy. In Group I, mutations are observed within the Kirsten rat sarcoma viral oncogene homolog (*KRAS*), serine/threonine-protein kinase B-raf (*BRAF*), phosphatidylinositol-3,4,5-trisphosphate phosphatase (*PTEN*) and phosphatidylinositol 3-kinase (*PI3K*) genes [4]. These mutations are rarely found in Group II, in which mutations are more frequent in the genes coding for breast cancer Type 1 susceptibility protein (BRCA1), breast cancer Type 2 susceptibility protein (BRCA2) and cellular tumor antigen p53 [4]. The International Federation of Gynecology and Obstetrics (FIGO) has issued a more detailed classification of ovarian tumors into four stages [5]. At advanced stages of tumor development, including tumors of the FIGO III and IV stages, metastases into the peritoneal cavity are formed, and the ongoing inflammatory process is responsible for the accumulation of a large amount of fluid called ascites [6]. In this specific microenvironment, cells can transform and migrate, and blood vessels can develop. Differences in the various zones of this microenvironment, concerning acidity, oxygen saturation and concentrations of various substances excreted by the surrounding tissues, can cause lesions and mutations in cancer cells, resulting in increased tumor heterogeneity and thus great difficulties in treatment [7]. Unlike peripheral blood plasma, ascites may contain indicators of the neoplastic process very early [8]. The concentrations of many of these indicators have been found to be higher in ascites than in blood serum [6,9,10,11,12].

Typical ascites fluid contains various non-neoplastic cell lines, including fibroblasts, infiltrating leukocytes, macrophages, pericytes, neuroendocrine cells, endothelial cells and adipocytes [9,10]. Adipocytes are a particularly important line. In humans, as in most other mammals, there are four types of adipose tissue: white, brown, beige and pink [13,14,15,16]. They differ in composition, function and location. White adipose tissue is mainly composed of mature adipocytes, preadipocytes, fibroblasts, endothelial cells and macrophages [13,14]. It has been shown that adipose tissue is a very important endocrine organ responsible not only for energy storage, but also for maintaining the body homeostasis, including the modulation of the immune response [17].

The adipose tissue accumulated in the abdominal cavity can be one of the components of a rich microenvironment for the development of tumors and their metastases. Considering ascites, this tissue is a very important endocrine and immune organ. Adipocytes, immune response cells, fibroblasts and epithelial cells present in adipose tissue are sources of diverse cytokines, chemokines and adipokines [17,18]. Lehr et al. [18] reported on more than 600 active proteins produced by adipocytes that play an important role in controlling metabolism. These proteins are excreted and act in an auto- and paracrine manner, affecting adipogenesis, development and function of adipocytes, migration of immune cells into adipose tissue [17], cell growth and differentiation, as well as glucose and lipid transport [18]. Adipokines acting in an endocrine manner regulate energy generation, appetite and satiety, as well as energy metabolism of insulin-sensitive tissues such as the liver, pancreas, muscle and adipose tissue. Moreover, they participate in inflammatory processes in both proinflammatory and anti-inflammatory roles [11,19,20].

Among the numerous adipokines, adiponectin, interleukin 6 (IL-6), interleukin 8 (IL-8), monocyte chemotactic protein-1 (MCP-1) and tissue inhibitor of metalloproteinase-1 (TIMP-1) have been reported as cytokines secreted most abundantly by omental adipocytes [21]. As a result, these adipokines might be important components of the ovarian cancer microenvironment in the peritoneal cavity, exerting a significant influence on tumor development and metastatic processes. Taking this into account, the presented article aims at reviewing the current state of knowledge about the presence of the above-mentioned adipokines in the blood and ascites of patients with ovarian cancer. Positive and/or negative effects of adiponectin, IL-6, IL-8, MCP-1 and TIMP-1 on carcinogenesis, tumor development and metastasis, as well as on the efficacy of the treatment are described. We would like to draw the attention of the research community to the importance of these proteins in the diagnosis, prognosis and choice of therapy in patients with ovarian cancer. Additionally, we would like to present the collected data on the blood and ascites concentrations of these adipokines in ovarian cancer patients, which might be helpful in planning further research in this area.

## 2. Literature Search

**Figure 1:**
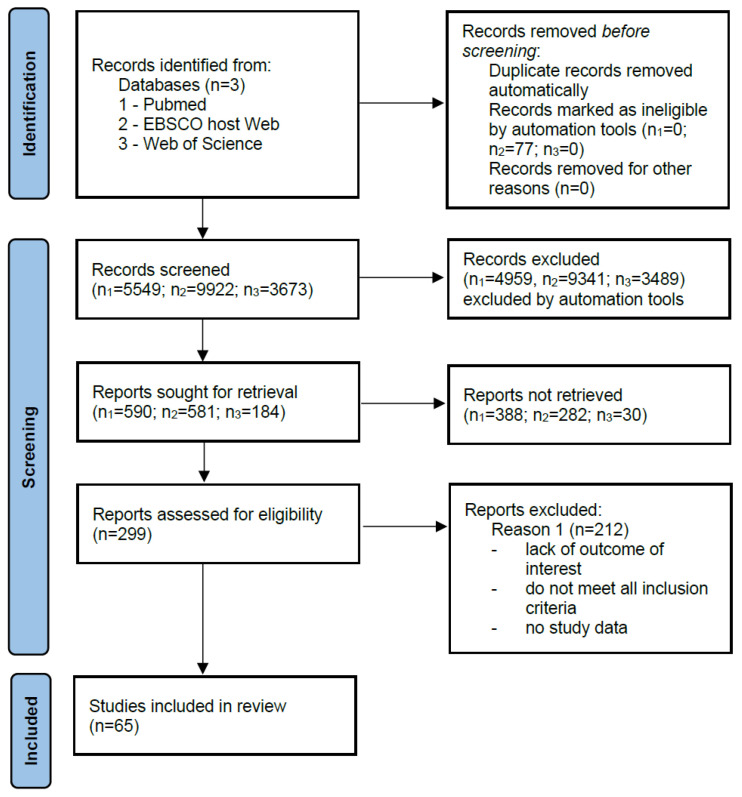
Literature search strategy. A total of 65 articles included studies of selected adipokines, of which 19 articles reported quantification of these proteins in blood and ascites.

There were no restrictions in collecting the data. The literature was reviewed in PubMed, Medline (EBSCO host Web), and Web of Science in the period from March 2020 to October 2020 to find data concerning the blood and ascites concentration of selected adipokines in ovarian cancer patients. Two investigators independently reviewed the reference list for potential eligible manuscripts and selected articles were then reviewed independently for inclusion in the analysis. The databases were searched for the following keywords: ascites, ovarian cancer, cytokines, adipokines, adiponectin, interleukin 6 or IL-6, interleukin 8 or IL-8, MCP-1, TIMP-1, concentration, level. No language restrictions were applied during the analysis. Articles that did not contain quantitative data on the analyzed proteins in the aspect of ovarian cancer with ascites were excluded. The records describing the quantification of the above-mentioned adipokines in blood (plasma/serum) and/or ascites were included (Figure 1).

From selected articles (*n* = 19), data were collected into a standardized form including publication year, source, number of cases, and type of adipokine characterized (Table 1) [22,23,24,25,26,27,28,29,30,31,32,33,34,35,36,37,38,39,40]. Other reported literature items discussed qualitative studies and general information on the selected adipokines as well as information on ovarian cancer and epidemiological data in the introduction.

## 3. Adiponectin

**Figure 2:**
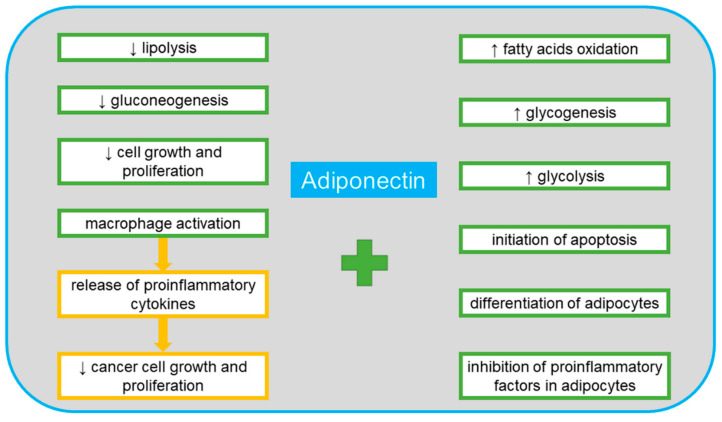
The positive systemic effects of adiponectin.

Adiponectin is one of the proteins produced by adipocytes. It is listed in the UniProt database under no. Q15848 [41]. Its alternative names include adipocyte complement-related 30 kDa protein (ACRP30), adipocyte, C1q and collagen domain-containing protein, adipose most abundant gene transcript 1 (apM-1) protein, ACDC, ADIPOQ and GBP28 [41]. It is composed of 244 amino acid residues, which, after cleavage of a 17-amino acid signal peptide, form a mature protein secreted into the bloodstream. The C-terminal (C1q) domain is a globular structure, and the N-terminal domain is a collagen-like structure [42]. The trimer presents in blood is one of the three possible adiponectin fractions. The other two are formed in a multimerization process, forming a medium molecular weight hexameric fraction and a high molecular weight fraction composed of 12–14 monomers [42]. The C1q domain can detach by proteolysis and form globular adiponectin with biological activity. This protein has important functions in the regulation of metabolism. Along with adiponectin receptor 2 (AdipoR2), it is involved in the activation of the fatty acid oxidation pathway. In combination with AdipoR1, it triggers the signal pathway leading not only to the oxidation of fatty acids, but also to vasodilation, increased rates of glycolysis and glycogenesis, the inhibition of lipolysis and gluconeogenesis, as well as increased cytoprotection by proteins such as the nuclear factor kappa-light-chain-enhancer of activated B cells (NF-κB) and phosphatidylinositol-3,4,5-trisphosphate phosphatase (PTEN) which lead to cell division arrest and initiation of apoptosis [43]. Adiponectin also has anti-diabetes, anti-inflammatory and anti-atherosclerotic functions [43]. It has been proven to play a direct role in improving the sensitivity to insulin at the whole-body level [44]. Adiponectin has a high degree of expression in adipose tissue. It affects the differentiation of adipocytes in an autocrine manner. An important role of adiponectin, especially in terms of neoplastic diseases, is its wide participation in inflammatory processes. This role is based on its paracrine effect on adipocytes by inhibiting proinflammatory agents and its endocrine effect on the polarization of macrophages [45]. Activation of macrophages into phenotype M1 enables them to release proinflammatory cytokines such as interleukin 1β (IL-1β) IL-6, interleukin 12 (IL-12) and tumor necrosis factor alpha (TNF-α) M2 macrophages induce the immune response by releasing anti-inflammatory cytokines. The anti-tumor activity of thus activated macrophages is based on the inhibition of cell growth and proliferation [11,22,45,46]. Macrophages with M1 and M2 phenotypes can be transformed into each other depending on the microenvironment and its activating molecules, secreted both by the macrophages themselves and by surrounding cells, including cancer cells [47]. Various transcription factors are involved in the polarization mechanisms of macrophages, such as the signal transducer and activator of transcription (STATs), peroxisome proliferator-activated receptor (PPAR)-γ, nuclear factor (NF-κB), interferon-regulatory factor (IRFs), activator protein (AP) 1, and cAMP-responsive element-binding protein (CREB). Adiponectin inhibits the production of TNF-a and IL-6 by reducing the NF-κB translocation into the cell nucleus [48]. As a result, the anti-inflammatory IL-10, TGF-β and the IL-1 receptor antagonist (IL-1RA) are induced. These factors stimulate macrophages to transform into the M2 population. It has been found that with a decrease in adiponectin levels, the pro-inflammatory activity increases, and conversely, with high levels of adiponectin, the nature of the immune response becomes anti-inflammatory [49]. In the aspect of neoplastic diseases, the macrophage M1 phenotype plays an anti-tumor role in the initiation of tumors, while the M2 phenotype promotes the development and metastasis of tumors [50]. Figure 2 shows the positive effects of adiponectin on the body.

Many researchers have been involved in the role of adiponectin in the development of cancer, including ovarian cancer, especially because this type of cancer is virtually asymptomatic until the FIGO III or IV stages, and there are still no specific markers for its diagnostics and early detection [51]. Studies have also shown a relationship between obesity and increased risk of ovarian cancer, although mechanisms linked to the role of excessive body mass in tumor formation have not been fully explained [52,53].

## 4. Adiponectin in Ovarian Cancer

Because of the apparent association of adiponectin with ovarian cancer, the level of this adipokine in blood serum as well as in ascites constituting tumor microenvironment could provide diagnostically important information. However, in the published studies, there are scarce data showing adiponectin serum levels in ovarian tumors, especially in comparison with its levels in ascites [22,23].

In the study of Jin et al. [23], adiponectin concentrations were determined in the blood plasma of 21 FIGO I/II patients and 31 FIGO III/IV patients [23]. Mean concentration in each study group was lower than in the control group. No statistically significant differences in adiponectin levels were found between earlier (FIGO I/II) and advanced metastatic (FIGO III/IV) stages, as well as between adiponectin level and BMI, and between adiponectin and other adipokine leptin [23]. Serum adiponectin concentrations were also determined in 43 patients with ovarian cancer before and after chemotherapy [22]. Mean concentration was lower before than after chemotherapy, with a difference of approximately 17.44%. In addition, leptin level and the leptin/adiponectin (L/A) ratio were determined. Although no significant correlation was found between disease stage or response to treatment and the concentrations of adiponectin and leptin, it was noted that lower L/A ratio before treatment was associated with a better response to chemotherapy [22].

Interestingly, according to Feng et al. [33], the levels of adiponectin expression in blood serum and in ascites were significantly higher in patients with ovarian cancer compared with the control group. In the blood serum of patients in the FIGO IV group, expression was higher than in those of the FIGO I group. No significant differences were found between the other types of ovarian cancer development among the studied FIGO I, II, III and IV groups. In the ascites of patients in the FIGO III (~7200.00 ± 300.00 ng/mL) and IV groups (~7700.00 ± 600.00 ng/mL), the level of adiponectin expression was higher in a statistically significant manner compared with the other two disease stages (values not provided) [33].

To conclude, most of the data available in the literature [22,23] demonstrate that blood plasma/serum adiponectin concentrations in patients with ovarian cancer are significantly lower than in control groups. However, an increase in adiponectin levels in the blood and ascites of patients with ovarian cancer was also observed [33]. The studies conducted so far on the molecular mechanisms of adiponectin action support its potential positive role in the process of carcinogenesis, tumor development and metastasis. Nonetheless, studies of the concentrations of this adipokine in cancer indicate that it may act differently in different neoplasms [33]. Few studies on adiponectin in the blood and ascites in ovarian cancer do not allow drawing definitive conclusions about the role of this adipokine in this neoplastic disease. Further intensive research carried out on a sufficiently large study group seems necessary to better understand the role of adiponectin in the development of ovarian cancer, as well as determine its potential role in diagnosis, prognosis and treatment. Table 2 shows concentrations of adiponectin in blood plasma or serum published in the literature.

## 5. Interleukin 6

**Figure 3:**
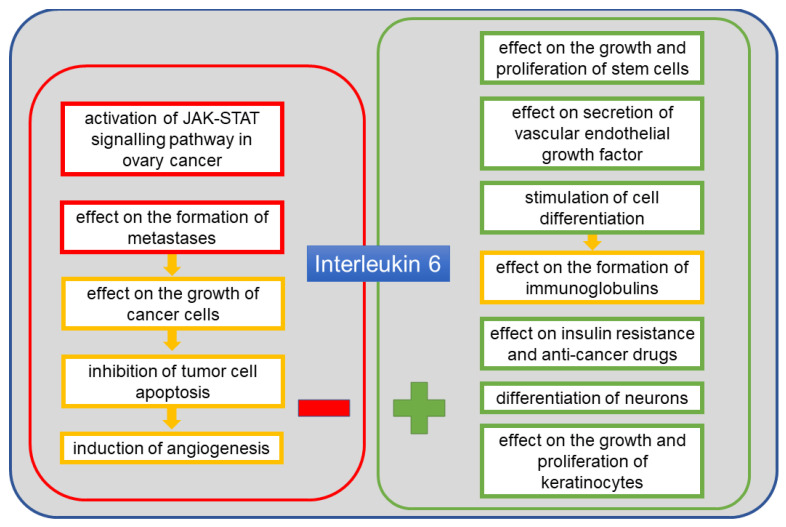
The positive and the negative systemic effects of interleukin 6 (IL-6).

IL-6 is another protein detected in the course of cancer, including ovarian cancer. It is a cytokine listed in the UniProt base under no. P05231 [41]. Its alternative names include B-cell stimulatory factor 2 (BSF-2), CTL differentiation factor (CDF), hybridoma growth factor, and interferon beta-2 (IFN-beta-2) [41]. This protein is composed of 212 amino acid residues and has a molecular weight of 23.7 kDa [41]. It is produced mainly by monocytes and macrophages, but also by fibroblasts, endothelial cells, T and B lymphocytes, keratinocytes, chondrocytes and adipocytes [54]. Among factors inducing the release of IL-6, IL-1, TNF-α, lipopolysaccharide, interferons and viruses should be mentioned. The effects of IL-6 activity include stimulation of cells capable of differentiating into immunoglobulin-producing cells, stimulation of growth and proliferation of keratinocytes and stem cells, differentiation of neurons, and vascular endothelial growth factor (VEGF) secretion. In the context of cancer, it facilitates tumor growth via mechanisms which inhibit apoptosis and induce angiogenesis [54,55]. It has been found that the level of circulating IL-6 produced by adipose tissue increases in obesity [14]. It is suggested that adipose tissue is an important source of IL-6 in humans, with a clear link between visceral fat and insulin resistance or inflammation [56]. A correlation between IL-6 levels in blood and ascites in the course of cancer, including ovarian cancer, has been observed in various studies [37,39,40]. The level of this cytokine is associated with tumor size, volume of ascites, progression of the disease and shorter survival. By influencing angiogenesis, it contributes to the spread of the tumor in the ascites microenvironment, resulting in poor prognosis and shorter survival [12,34]. IL-6 level has also been associated with resistance to anticancer drugs. The contribution of IL-6 to the neoplasia of ovarian cancer is mainly due to the potential overactivation of the Janus kinases and signal transducers and activators of transcription factor (JAK–STAT) signaling pathway [57]. Overexpression of the signal transducer and activator of transcription 3 factor (STAT3) protein has been confirmed in ovarian cancer cells [57]. Figure 3 shows the positive and the negative effects of IL-6 on the body.

## 6. IL-6 in Ovarian Cancer

In a study by Lane et al. [34] in 39 patients with ovarian cancer, the mean concentration of IL-6 was determined in ascites. There were statistically significant differences between the levels of this interleukin depending on the FIGO stage, histopathological diagnosis and the serum levels of the cancer antigen 125 (CA125) glycoprotein. Median progression-free survival was 14 months in patients with higher IL-6 levels and 24 months in patients with lower levels of this interleukin. As IL-6 is involved in tumor angiogenesis, it affects the development and progression of the tumor by promoting the development of ascites [34]. Therefore, measurement of the concentration of this adipokine in ascites seems more justified than measurement of its concentration in blood serum from which the protein can be cleared more rapidly [34]. In another study by Lane et al. [35] in 53 patients diagnosed with serous epithelial ovarian cancer classified as FIGO III and IV, the concentration of selected cytokines in ascites was determined. The control group in this study consisted of 10 women with benign gynecological conditions. IL-6 level in the control group was significantly lower than in patients with advanced ovarian cancer. In addition, the level of IL-6 determined in ascites was correlated with the serum level of the CA125, and 92% of patients with malignant ovarian tumors had CA125 > 35 UI/mL and IL-6 > 45 pg/mL. In contrast, none of the patients with benign gynecological conditions reached such high levels of the combination of the two markers. CA125 marker is not just specific to cancer but also to benign gynecological problems such as endometriosis, as well as liver disease or pregnancy. Along with IL-6, it forms a tandem helpful in the diagnostics of cancer, including ovarian malignancies—serous epithelial ovarian cancer [35]. In a study conducted in 74 patients aged 18 to 89 years, minimum IL-6 concentration was determined in ascites [36]. Patients were divided into two subgroups: Group 1 including 36 patients with malignant ovarian tumors (serous carcinoma of various histopathological types) and Group 2 including 38 patients with benign gynecological conditions (ovarian cysts, benign epithelial tumors, endometrioid cysts). In Group 1, the mean concentration of IL-6 in ascites was higher than in Group 2. Considering the FIGO staging, it was found that the mean concentration of IL-6 was significantly lower in FIGO I and II groups than in FIGO III and IV groups. Tumor spread in the peritoneum and the presence of tumor cells in ascites contributed to the increase in IL-6 level. Therefore, after analyzing all correlations between the proteins studied and the type and stage of disease, it was concluded that IL-6 is a cytokine of a high diagnostic value [36]. Kampan et al. [37] examined 33 women aged 41 to 83 years diagnosed with obesity and serous ovarian cancer, mainly at the FIGO III stage (two cases of FIGO IV ovarian cancer were also included). The levels of IL-6 in blood serum and ascites were determined and compared with those of a group of 12 obese women aged 38 to 73 years with benign ovary conditions and a group of 21 obese women aged 40 to 84 years without ovary conditions. Using a multiplex panel of 28 markers, the highest IL-6 concentration in blood serum was obtained in patients with advanced ovarian cancer, lower for patients with benign ovary conditions and the lowest for women with no such conditions. It was found that the IL-6 level in 28.5% of patients with no ovary conditions and 6.7% with benign conditions was below the detection limit of the measurement method used. Of 33 women with advanced ovarian cancer, ascites was found in only 18. The concentration of IL-6 in this group was significantly higher in ascites than in blood serum. No correlation was found between the higher serum IL-6 concentration and the presence of ascites. However, the authors stressed the importance and promising potential of this marker in combination with, e.g., the CA125 in pre-operative evaluation of suspected ovarian tumors, in order to distinguish malignant or benign ovary lesions from normal ovaries [37]. In a similar study, Matte et al. [38] determined the concentration of IL-6 in the ascites of 38 patients aged 27 to 85 years with epithelial ovarian cancer at the FIGO I (8 cases), FIGO II (4 cases), FIGO III (17 cases) and FIGO IV (9 cases) stages. The assay was performed using a multiplex panel of 120 cytokines for 10 selected patients and the ELISA method for the whole study group. The authors did not report the results of the multiplex cytokine panel, but declared a strong correlation between the results obtained using both methods. In a comparison of IL-6 concentrations in correlation with cancer stage, no statistically significant differences were found between FIGO I/II and FIGO III/IV groups [38]. Canton-Romero et al. [39] examined 21 patients aged 34 to 73 years, divided into three groups depending on their response to carboplatin chemotherapy. Group 1 included 10 women with platinum-sensitive FIGO I (2 cases), FIGO II (2 cases) and FIGO III (6 cases) cancer. Group 2 included four women with platinum-resistant FIGO III (3 cases) and FIGO IV (1 case) cancer. Group 3 comprised seven women with FIGO III (5 cases) and FIGO IV (2 cases) cancer with primary resistance to platinum. A significantly higher level of IL-6 was found in the ascites of all patients compared with the blood plasma of the 6 control group participants. Comparison of IL-6 concentrations in the three groups of patients with different sensitivity to carboplatin did not reveal any significant differences between patient groups and between each of these groups and the control group (6 healthy women). No significant differences were found for IL-6 concentrations in both blood plasma and ascites. However, according to the authors, the results of IL-6 concentration measurements show an important role of this cytokine in ovarian cancer, associated with the development of a specific tumor microenvironment in ascites, owing to the correlation with poor prognosis and inadequate response to treatment [39]. Maccio et al. [40] studied 104 patients with FIGO I and II (29 patients) and FIGO III and IV (75 patients) ovarian cancer for IL-6 concentration in blood serum. Control group consisted of 95 healthy women. Mean concentrations of IL-6 in blood serum in the control group and in patients with FIGO I and II cancer were significantly lower than in patients with FIGO III and IV cancer. No statistically significant differences were observed between the control group and patients with early-stage cancer (FIGO I and II). The authors stressed the linear relationship between the concentrations of IL-6 and leptin, reactive oxygen species and glutathione peroxidase. The authors concluded that IL-6 and leptin can play the role of early markers of metabolic changes associated with cancer development. Assessing these markers could allow providing patients with more appropriate care and implementation of best possible therapies [40]. Giuntoli et al. [24] tested a panel of 22 cytokines, including IL-6, in 37 patients with advanced ovarian cancer (Stages III and IV). The levels of IL-6 obtained in ascites were significantly higher compared with blood plasma concentrations [24]. In a study of 8 patients aged 68 to 78 years, treated for FIGO IIIC and IV serous ovarian cancer, the concentration of IL-6 determined in ascites was not significantly different from that in the control group—8 women with benign ovary conditions, age-matched to the study group [25]. In contrast to the concentrations of adiponectin, those of IL-6 are reported in the literature for ascites in ovarian cancer. Data on IL-6 concentrations collected in Table 3 allow a conclusion that in control groups, comprising patients with benign gynecological conditions, IL-6 concentrations were significantly lower than in patients with ovarian cancer. Differences in mean concentrations are certainly due to the specificity of individual study groups. These groups varied in both the number and type of benign gynecological conditions.

Correspondingly, values of IL-6 concentrations in blood plasma/serum presented in Table 4 also show pronounced differences between studies. However, consistently higher concentrations are observed in ovarian cancer, lower in benign gynecological conditions (e.g., endometriosis) and the lowest in healthy patients. The research on the molecular mechanisms of IL-6 activity indicates a negative role of this cytokine in the process of carcinogenesis, tumor development and metastasis. Numerous studies of the concentration of IL-6 in the blood and ascites of patients with ovarian cancer confirm increased levels of this protein in neoplastic disease and the correlation of IL-6 secretion with the advanced stage of the disease. It should be emphasized that the concentration of IL-6 in ascites is significantly higher than in blood, which indicates an elevated secretion of this cytokine in the microenvironment of the ovarian tumor. Of particular interest is the possibility of simultaneous measurement of IL-6 and CA125 in order to obtain a more precise diagnosis regarding the patient’s condition, prognosis and evaluation of the therapy used. Taking into account the current state of knowledge, further studies can be strongly recommended in order to transfer the results of the existing studies on IL-6 in ovarian cancer to clinical use.

## 7. Interleukin 8

IL-8 is a chemotactic protein released in response to inflammatory stimuli. This chemokine (UniProt no. P10145) is known under multiple names: C-X-C motif (chemokine 8), emoctakin, chemokine (C-X-C motif) ligand 8 (CXCL8), granulocyte chemotactic protein 1 (GCP-1), monocyte-derived neutrophil chemotactic factor (MDNCF), monocyte-derived neutrophil-activating peptide (MONAP), neutrophil-activating protein 1 (NAP-1), protein 3-10C, and T-cell chemotactic factor [41]. It is encoded by the *CXCL8* gene and comprises 99 amino acid residues. Following cleavage of the 20-amino acid (aa) signal sequence and as a result of post-translational N-terminal modifications, several isoforms with different activity are released from cells. The name IL-8 refers to the 77-aa isoform that occurs in non-immune cells [41]. Other isoforms include the 79-aa CDNCF-a, the 73-aa IL-8(5–77), the 72-aa monocyte and macrophage IL-8(6–77), the 71-aa IL-8(7–77), the 70-aa IL-8(8–77) and the 69-aa IL-8(9–77) [41].

**Figure 4:**
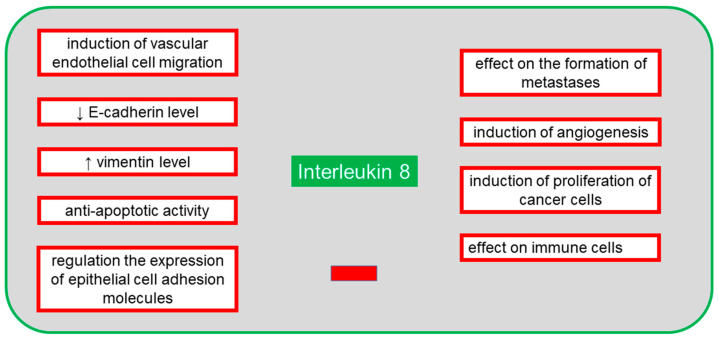
The negative systemic effects of interleukin 8 (IL-8) on the body in the presence of an ongoing neoplastic process.

The protein is secreted by several cell types, including neutrophils, monocytes, mesothelial and endothelial cells, as well as neoplastic cells [58]. In the case of ovarian cancer, IL-8 is present in peritoneal fluid, ovarian cyst fluid, blood serum and tumor tissue. According to many authors, the increased expression of IL-8 in cancer patients is correlated with poor prognosis and chemosensitivity [58,59]. Pathological overexpression of many cytokines, including IL-8, promotes cancer metastasis, and its high level persists throughout the course of the disease [26]. IL-8 is activated by binding with CXCR1 and CXCR2 receptors, and thus activated protein exerts its function via immune cells and chemotaxis. A study of CXCR1 and CXCR2 activation by IL-8 and inhibition by reparixin confirmed that CXCR2 was primarily present on ovarian cancer cells, and its high expression was strongly associated with shorter survival and increased metastatic potential [60]. It appears that both exogenous and endogenous IL-8 leads to a decrease in cadherin E and an increase in vimentin [61]. These proteins allow the tumor cell to develop a mesenchymal phenotype in which the cell has increased mobility, does not communicate with other cells and, therefore, has a greater capacity to migrate and form metastases [60]. IL-8 expression can be controlled by proteins such as NF-κB, TNF-α, IL-1β, chemical and environmental stimuli and steroid hormones [61]. Expression of IL-8 receptors in neoplastic cells, endothelial cells, neutrophils and macrophages shows that the autocrine and paracrine activities of IL-8 have a significant effect on tumor microenvironment by inducing multiple signal pathways contributing to angiogenesis, proliferation and migration of endothelial cells, survival and regulation of neutrophil infiltration into the microenvironment, which promotes metastatic activity [61]. IL-8, together with IL-6, participates in the paracrine mechanism of expression regulation of the epithelial cell adhesion molecule (EpCAM), which contributes to tumor growth and whose overexpression occurs in ovarian cancer cells [62]. In this poorly known mechanism, IL-8 activates the EpCAM expression, while IL-6 is an inhibitor of IL-8. It is known that therapy using the tumor necrosis factor-related apoptosis-inducing ligand (TRAIL) sensitizes neoplastic cells to induced apoptosis which is dependent on the membrane receptors TRAIL-R1/DR4 and TRAIL-R2/DR5 and on the presence of intracellular pro- and anti-apoptotic proteins [63]. As IL-8 is anti-apoptotic, and TRAIL is pro-apoptotic, the presence of IL-8 in ovarian cancer cells and microenvironment complicates or even prevents therapeutic use of TRAIL [63]. Figure 4 shows the negative effects of IL-8 on the body in the presence of an ongoing neoplastic process.

## 8. IL-8 in Ovarian Cancer

In a study by Sanguinete et al. [27], 26 patients (mean age of 52 years) with FIGO I–III ovarian cancer were examined. IL-8 concentration in blood serum in 21 patients with FIGO I–IIIB cancer was significantly lower than in 5 patients with FIGO IIIC disease. There was no correlation between IL-8 concentration in 16 patients with overall survival ≤ 60 months and 8 patients with overall survival > 60 months. However, the study showed a correlation between higher serum IL-8 level and the ratio of neutrophils to lymphocytes ≥ 4, the ratio of thrombocytes to lymphocytes ≥ 200 and altered level of CA125, and the FIGO IIIC stage of disease, which puts IL-8 as a marker of poor prognosis in ovarian cancer [27]. A group of 156 patients in two age groups, ≤50 and >50 years were also studied, including 92 with malignant ovarian tumors, 64 with benign tumors and 58 healthy participants (a control group) [26]. Among patients with malignant tumors, 20 had FIGO I–II cancer and 72 had FIGO III–IV cancer. The lowest IL-8 concentration in blood serum was observed in the control group, higher values were observed in the patients with benign tumors, and the highest values were observed in the patients with malignant ovarian cancer, with differences between these three groups being statistically significant. A significantly lower level of this protein was found in the 20 patients with FIGO I and II cancer compared with the 72 patients with FIGO III and IV cancer. In addition, correlation of IL-8 concentration and the presence or absence of metastases after chemotherapy was tested. In 59 patients with metastases and 33 patients with stable disease after chemotherapy, IL-8 level was lower than in the entire group of 92 patients with ovarian cancer before chemotherapy. The study showed the effect of high expression of IL-8 on the occurrence and development of ovarian cancer and on its sensitivity to chemotherapy; moreover, changes in the level of this protein were found to affect prognosis and response to treatment [26]. In a study in 21 patients with ovarian cancer, significantly lower level of IL-8 in blood serum than in ascites was found [28]. The authors stressed that because of the high level of IL-8 in ascites, inflammation played an important role in the course of ovarian cancer [28]. Farkas et al. [64] carried out a comparative analysis of IL-8 concentrations in blood serum and ascites in 16 patients with ovarian hyperstimulation syndrome, 16 patients with serous ovarian cancer in Stages IIIB or IIIC and 17 patients with endometriosis. Control group comprised 21 patients with benign ovary conditions from whom blood samples were collected, and intraoperative pelvic washing was performed during laparoscopic cystectomy. The authors did not report specific concentrations but declared higher concentration of IL-8 in ascites than in blood serum. By analyzing the concentrations of this protein in ascites in correlation with diagnosis, the authors revealed significantly higher values in patients with ovarian cancer and ovarian hyperstimulation syndrome. A positive correlation was also determined between the concentrations of IL-8 and IL-10, and between VEGF and transforming growth factor β (TGF-β). The study groups differed in the number and mean age of patients, which clearly affected the inflammation profile. However, a trend toward production of cytokines in ascites was found, suggesting the same pathomechanism of developing ascites in both ovarian cancer and ovarian hyperstimulation syndrome or other benign ovary conditions [64]. A study to determine IL-8 concentrations in ascites, blood serum and tumor tissue from 35 patients aged 41 to 78 years with ovarian cancer at the FIGO III and IV Stages was conducted [29]. The statistically lowest level of IL-8 was found in blood serum, and the highest in tumor tissue [29]. In a study in 8 patients aged 68 to 78 years, treated for FIGO IIIC and IV serous ovarian cancer, mean concentration of IL-8 was determined in ascites in comparison with the control group comprising 8 women with benign ovary conditions, age-matched with patients in the study group [25]. No statistically significant differences were observed. However, mesothelial cells of the peritoneal cavity that were in contact with ascites showed an increased rate of aging along with a decreased rate of proliferation. These cells stimulated further release of IL-8, which had an impact on increased migration of cancer cells; hence, they had a pro-cancerogenic activity which promoted metastasis [25]. Based on the literature data on IL-8 concentrations in ascites presented in Table 5, it was found that its levels in advanced ovarian tumors are higher than in benign gynecological conditions [25,28,29].

Based on the analysis of the presented data (Table 6) on the levels of IL-8 in blood serum, it can be suggested that the concentration of this protein increases along with the stage of ovarian cancer. Lower values were observed in control groups with healthy participants, and higher in patients with ovarian cancer at Stages III–IV. Compared with the concentrations in ascites, those in blood serum are lower or similar [26,27,28,29].

In the case of IL-8, the results of the studies conducted so far concomitantly indicate higher concentrations of this cytokine in the blood of patients with ovarian cancer. Elevated levels of this cytokine have also been found in ascites of ovarian cancer patients. Noteworthy is the large dispersion of the concentration of this adipokine measured in both blood and ascites in various studies. However, it seems to be confirmed that the molecular mechanisms of action of IL-8 promote carcinogenesis, tumor development and metastasis. Since the highest values of IL-8 are found in advanced stage of ovarian cancer, it seems that the level of this adipokine could be determined as an additional parameter supporting the assessment of treatment efficacy and prognosis in patients with ovarian cancer. The participation of IL-8 in pathological processes in the tumor microenvironment should also be taken into account when planning new individualized therapeutic strategies and in research into new anti-cancer compounds. The possibility of using molecules with properties to inhibit the action of IL-8 might also be considered in order to increase the effectiveness of the treatment. 

## 9. Monocyte Chemotactic Protein-1 (MCP-1)

The chemokine MCP-1 is another protein involved in cellular pathways. MCP-1 was the first human CC chemokine discovered [65]. It belongs to small heparin-binding proteins. It is listed in UniProt under no. P13500, and its recommended name is C-C motif chemokine 2 [41]. The allowed names include HC11, monocyte chemoattractant protein 1, monocyte chemotactic and activating factor (MCAF), monocyte chemotactic protein 1 (MCP-1), monocyte secretory protein JE, small-inducible cytokine A2 [41]. The protein is encoded by the CCL2 gene (synonyms: MCP1, SCYA2) and composed of 99 amino acid residues, of which 23 amino acids constitute a signal peptide. The protein is expressed primarily in monocytes, but also in seminal fluid, endometrial fluid and vesicular fluid [41]. It is also produced by endothelial and epithelial cells, fibroblasts, smooth muscle cells, astrocytes and microglial cells [65]. MCP-1 is produced via induction by other cytokines, growth factors or oxidative stress [65].

**Figure 5:**
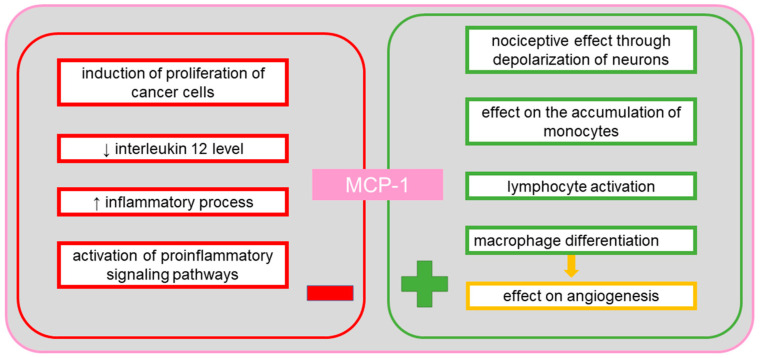
The positive and the negative systemic effects of monocyte chemotactic protein-1 (MCP-1).

MCP-1 plays an important role in many diseases, including tuberculosis, by reducing the IL-12 level, in multiple sclerosis via a correlation mechanism between MCP-1 and axon damage, as well as between MCP-1 and interferon gamma-induced protein 10 (IP-10) in hypertrophic astrocytes, in neovascularization of tumors via infiltrating macrophages, in inflammatory bowel disease by influencing the differentiation of intestinal macrophages, and demonstrates nociceptive activity via its effect on the depolarization of neurons [65]. In cancer, MCP-1 stimulates protective processes by attracting and activating lymphocytes, and is responsible for tumor progression owing to angiogenic activity. The MCP-1 protein, which is released by macrophages and regulatory T cells (Treg), regulates the expression of the endothelial growth inhibitor—tumor necrosis factor ligand superfamily member 15 (TNFSF15), causing a shift in the angiogenic balance in the tumor microenvironment toward inflammatory processes and neovascularization [66]. Thus, the MCP-1 protein is a potent marker of tumor progression [66]. A study in mice established the importance of MCP-1 and the C-C chemokine receptor type 2 (CCR2) in the accumulation of monocytes during inflammation [67]. In ovarian tumors, the two molecules become significant markers of the degree of infiltration by macrophages in the changed tissue [67]. CCR2 for MCP-1 occurs in two isoforms known as A and B [67]. In monocytes and NK cells, the B isoform is mainly present. It has been established that CCR2 recognizes not only MCP-1, but also other proteins of this family of chemokines, including MCP-2 and MCP-3 [67]. Many studies have suggested the effect of proinflammatory and anti-inflammatory signals on the regulation of expression of the gene encoding the chemokine receptor in human phagocytes [68]. The expression of the receptor is also regulated by the pro-neoplastic TNF-α [69]. It has been found that proinflammatory lipopolysaccharide decreases the level of MCP-1 and other cytokines, such as IL-6, IL-10 and VEGF, in ascites and inhibits the expression of the chemokine receptor [70]. Inhibition of receptor expression can be used as a stop signal, leading to the retention of phagocytes at the inflammation site [69]. In a study of a serous ovarian cancer cell line, a concentration-dependent effect on cell invasion was observed [70]. Additionally, high concentrations caused adhesion of cells. Following the use of CCR2 antagonists, the degree of invasion and adhesion of cancer cells was observed to be reduced [70]. Moreover, it was noted that the effect of MCP-1 on invasion and adhesion was independent of M2d macrophages (TAMs—tumor-associated macrophages), which play a role in tumor formation by activation of proinflammatory intracellular pathways, transformation into a form which inhibits signaling routes associated with cytokine expression, stimulation of angiogenesis and migration enabling metastasis [70]. In patients with epithelial ovarian tumors, increased MCP-1 expression has been observed in ascites [71]. In mouse studies, treatment with dabigatran, a thrombin inhibitor, has been found to reduce the MCP-1 level in ascites [71]. Because incubation with thrombin increases the release of MCP-1, use of dabigatran allows reducing the level of MCP-1 in cancer microenvironment [71]. MCP-1 is also a chemotactic factor for NK cells (CD56+/CD16+), but the presence of these cytotoxic and immunoregulating cells is rarely observed in ovarian cancer [72]. In contrast, CCR2 expression has been detected in CD3+ and CD4+ T-cells and in CD14 + macrophages found in ascites in ovarian cancer [73]. Figure 5 shows the positive and the negative effects of MCP-1 on the body.

## 10. MCP-1 in Ovarian Cancer

In a study in 8 patients aged 68 to 78 years, treated for FIGO IIIC and IV serous ovarian cancer, mean concentration of MCP-1 in ascites was not significantly different from that in the control group comprising 8 age-matched women with benign ovary conditions [25]. Elevated MCP-1 level could affect tumor progression and metastasis similarly to IL-8 [25]. Hefler et al. [30] carried out extensive studies in which they determined blood serum MCP-1 concentrations in four groups of patients aged 29 to 87 years. Group 1 included 8 patients with FIGO I, 11 patients with FIGO II and 29 patients with FIGO III ovarian cancer. Group 2 included 38 patients with relapsing ovarian cancer. Group 3 included 67 patients with benign ovary conditions, while control group included 45 healthy women. The study demonstrated statistically significant differences between blood serum concentrations of MCP-1 in patients with malignant and relapsing cancer (Groups 1 and 2 combined), and patients with benign ovary conditions and the control group (Group 3 and control combined). The highest levels of MCP-1 were obtained for malignant cancer, then for relapsing disease, for benign ovary conditions and for healthy participants. In addition, the authors stressed the role of MCP-1 in the differentiation of malignant neoplasms from benign adnexal masses, as a marker auxiliary to the blood serum level of CA125 [30]. The MCP-1 protein was isolated and purified using a four-step process from ascites obtained from 12 patients with epithelial ovarian cancer at the FIGO Stages IC–IV and 12 patients with non-ovarian tumors, including 3 with benign ovary conditions and 9 with non-gynecological cancers [31]. The observed concentrations of MCP-1 did not show statistically significant differences between the studied groups of patients with malignant ovarian cancer and those with benign ovary conditions and non-gynecological cancers [31].

Table 7 shows the concentrations of MCP-1 in ascites. No statistically significant differences were found in patients with advanced ovarian cancer compared with those with benign gynecological conditions.

The more advanced the disease, the higher the concentration of MCP-1 in blood serum. Statistically significant differences reported in the literature are presented in Table 8.

In conclusion, it can be assumed that MCP-1 might be a useful additional parameter measured in the blood of patients with ovarian cancer to obtain a more precise diagnosis, especially in combination with CA125. Increased MCP-1 levels might be related to a higher risk of tumor progression and a worse prognosis. On the other hand, there were no differences in the MCP-1 concentration in the ascites of patients with ovarian cancer and benign gynecological conditions. It can be suggested that MCP-1 does not play an important role in the peritoneal ovarian tumor microenvironment. Nevertheless, further research should focus on assessing the possibility of using MCP-1 as an auxiliary marker of advanced ovarian cancer. 

## 11. Tissue Inhibitor of Metalloproteinase-1 (TIMP-1)

**Figure 6:**
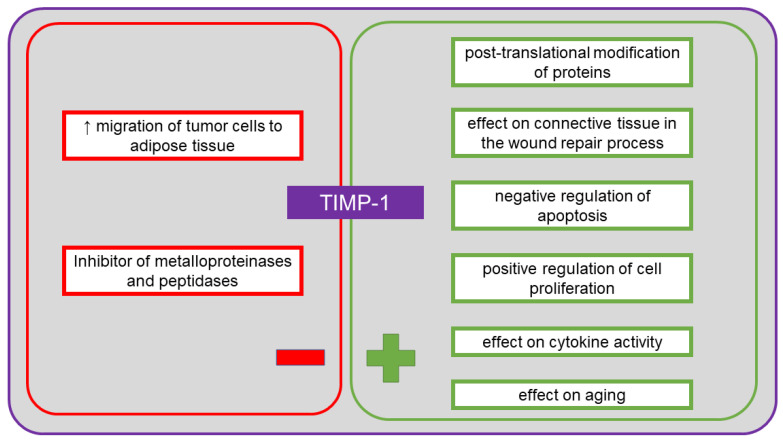
The positive and the negative systemic effects of tissue inhibitor of metalloproteinase-1 (TIMP-1).

TIMP-1 is a tissue inhibitor of metalloproteinases endowed with cytokine activity, also involved in neoplasia. It is a glycoprotein listed in UniProt under no. P01033 [41]. The recommended name is metalloproteinase inhibitor 1, and allowed aliases include tissue inhibitor of metalloproteinase-1 (TIMP-1), erythroid-potentiating activity (EPA), fibroblast collagenase inhibitor, collagenase inhibitor [41]. In humans, it is encoded by the *TIMP1* gene (*CLGI, TIMP*) [41]. TIMP-1 consists of 207 amino acid residues, of which the first 23 form a signal peptide. Among the identified functions of this protein, in addition to its cytokine activity, there are activity as a growth factor, inhibitor of metalloproteinases and peptidases, as well as binding agent for proteases and zinc ions [41]. It is involved in several biological processes, including aging, cartilage formation, cell protein metabolism, replacement of connective tissue involved in wound healing in response to inflammatory process, signaling pathways involving cytokines, negative regulation of apoptosis, positive regulation of cell proliferation, post-translational protein modification, response to cytokines, hormones and organic substances [41]. TIMP-1 plays an important role in the regulation of metalloproteinase activity. The protein inhibits the activity of proteases by forming a permanent and irreversible non-covalent complex [74]. Homolog 1 of this inhibitor can inhibit numerous matrix metalloproteinases (MMPs), including collagenases (MMP-1), stromelysins (MMP-3) and gelatinases (MMP-9) [74]. Overexpression of MMP-9, together with the imbalance between MMP-9 and TIMP-1, play an important role in the development of ovarian cancer, when the blood serum level of TIMP-1 is elevated [74]. During the formation of metastases of ovarian cancer, TIMP-1 can be most commonly detected in the omentum [73]. Adipocytes in the omentum produce several types of cytokines, including IL-6, IL-8 and TIMP-1, which facilitate the migration of neoplastic cells into the adipose tissue of the omentum [73]. It has been suggested that limited expression of TIMP-1 in the blood serum of patients after chemotherapy is a good prognostic factor for patients with ovarian cancer, although it is not entirely clear whether the inhibition of TIMP-1 expression in the neoplastic tissue of such patients may indicate the same prognosis [75]. It has also been shown that the expression of MMP-9 and TIMP-1 induced by the epidermal growth factor (EGF) in ovarian cancer cell lines results in increased migration and mass spread induced by EGF [76]. Studies have shown that lysophosphatidic acid limits the effect of metalloproteinase inhibitors, including TIMP-1, TIMP-2 and TIMP-3 [77]. In metastatic ovarian tumors, a microenvironment is formed by peritoneal fluid, and a higher level of lysophosphatidic acid has been observed in this fluid [77]. Lysophosphatidic acid stimulates the spread of primary tumor cells, but not of epithelial ovarian cancer or borderline tumors [77]. A study by Sonego et al. [78] demonstrated that TIMP-1 is overexpressed in the cells of platinum-resistant epithelial ovarian cancer. Increased and maintained overexpression of this inhibitor can be induced by platinum-based therapies. It was suggested that TIMP-1 contributes to the development of resistance to platinum [78]. Therefore, it seems important to define the level of this marker to aid selection of the most effective therapy. As MMPs are involved in the degradation of extracellular matrix and in angiogenesis, they play a role in the spread of tumor cells and metastasis [32]. Metalloproteinase inhibitors, including TIMP-1, are regulators of the activity of the MMP proteins, but also have pro-neoplastic and pro-metastatic properties [32]. Increased expression is usually associated with poor prognosis, although the available studies of ovarian cancer indicate both downregulation and upregulation [79,80,81]. Figure 6 shows the positive and the negative effects of TIMP-1 on the body.

## 12. TIMP-1 in Ovarian Cancer

A comparison study of several plasma and ascites proteins was conducted in a group of patients with serous ovarian cancer [82]. The study involved 70 samples of ascites and 20 samples of blood plasma. In addition, 10 plasma samples from patients with benign ovary conditions were analyzed. The concentration of TIMP-1 in ascites in the course of serous ovarian cancer was 32.00 higher than in blood plasma [82]. In a study in 37 patients aged 29 to 78 years with epithelial ovarian cancer at the FIGO I (1 case), FIGO II (1 case), FIGO III (29 cases) and FIGO IV (6 cases) stages, concentrations of TIMP-1 in blood serum were determined [32]. Blood samples were collected from each patient four times. The first and second sample were obtained within seven days before and within seven days after a cytoreductive surgery, respectively. The third and fourth sample were obtained during chemotherapy (three weeks after the third cycle) and after chemotherapy (up to three months after treatment completion), respectively. TIMP-1 concentrations measured were the highest after the cytoreductive procedure. Lower values were obtained before the surgery, and the lowest during and after chemotherapy. Based on these correlations, reduced overall survival in patients with high TIMP-1 levels after chemotherapy was found. Moreover, higher TIMP-1 concentrations correlated with larger volumes of ascites. A high correlation was also identified between the TIMP-1 and VEGF levels for samples taken at each stage of the study, i.e., from pre-surgery to after chemotherapy. These correlations suggest common regulatory mechanisms for both ovarian cancer markers, leading to neovascularization and degradation of the extracellular matrix and basal membranes [32].

No quantitative data were found in the literature for the TIMP-1 levels in ascites in ovarian cancer. Table 9 presents TIMP-1 concentrations in blood serum obtained only from patients with advanced stages (FIGO III and IV) of ovarian cancer. Within this group, a trend of concentration changes is shown in relation to the treatment stage, i.e., before and after cytoreductive surgery as well as during and after chemotherapy. These scarce data indicate that the highest concentration occurs after the cytoreductive procedure and the lowest concentration occurs after a completed treatment [32].

It should be noted that TIMP-1 is a protein associated with the described role in the progression of ovarian cancer and associated with poor clinical outcomes. There are very limited data on blood levels of TIMP-1 and ascites in patients with ovarian cancer. However, it can be concluded that the decreased levels of this inhibitor after treatment are associated with a better prognosis for the patient. The very high level of TIMP-1 in ascites compared with blood plasma indicates the intensive secretion of TIMP-1 into the peritoneal cavity and its role in the ovarian tumor microenvironment. Further research in this area may provide a better understanding of the function of TIMP-1 in the development and metastasis of ovarian cancer, as well as resistance to chemotherapy. Similarly to the aforementioned adipokines, TIMP-1 may be an auxiliary marker used in the diagnosis, prognosis and evaluation of the effectiveness of therapy in patients with ovarian cancer.

The results found in the literature regarding the selected adipokines measured in blood and ascites are summarized in Table 10.

## 13. Conclusions

Inflammation factors are detected not only in blood but also in ascites and in tumor tissue. Numerous studies regarding various adipokines, a subset of cytokines, associated with the presence of tumor cells highlight the fact that the concentrations of these proteins are much higher in ascites than in blood serum/plasma. It was noted over 10 years ago that out of dozens of cytokines produced by omental adipocytes and affecting metastasis, five are the most abundant, namely adiponectin, IL-6, IL-8, MCP-1 and TIMP-1 [21,83]. This review discusses the levels of these five cytokines in ascites and in blood plasma/serum in the course of ovarian cancer. No specific data regarding the level of adiponectin in ascites were found for ovarian cancer. For IL-6 and IL-8, it can be confirmed that their concentrations in ascites in the course of ovarian cancer are higher than in blood plasma/serum. The trend is similar for benign gynecological conditions. The higher concentrations of these cytokines in the microenvironment surrounding the ovaries seem to be due to direct contact of the accumulating fluid with the adipose tissue cells. There is a scarcity of data regarding the concentrations of adiponectin and TIMP-1 in ascites in the course of ovarian cancer. On the one hand, multiple studies indicate an association between the level of TIMP-1 expression and poor prognosis in patients with ovarian cancer. On the other hand, some studies highlight a lack of correlation between the expression of TIMP-1 and/or TIMP-2 and overall survival in these patients. However, it is considered that the effect of TIMP-1 level on the prognosis is probably less pronounced than it was originally suspected [84]. As these cytokines are products of adipocytes, other factors will certainly affect their concentrations in addition to the presence of a developing tumor. These factors include, but are not limited to, the following: age, body mass, obesity, visceral fat content, ongoing inflammatory processes associated with cancer cell hyperplasia and metastasis and the type of cancer. However, it could be assumed that, in addition to the histological examination of the malignant cells found in the ascites fluid sample, examination of the adipokines in the same ascites fluid sample may provide additional information and help find a better and individualized therapy for the treatment of the patient. Determining the correct values that can help the diagnostics of ovarian cancer requires many studies with limited categories of patients included in study groups. Analysis of the available studies suggests that it is important to determine associations between the levels of different cytokines, and that the ratios of cytokine levels can be better markers for both diagnosis and treatment than the levels of individual cytokines. Systematic studies in this area could clarify the usefulness of determining the levels of cytokines as markers of cancer, its progression and prognosis. Consequently, this could enable earlier initiation and monitoring of adequate treatment.

## Figures and Tables

**Table 1:** Characteristics of quantitative studies included.

Study	Year	Journal	No of Cases	Adipokine
Słomian et al. [22]	2019	Endokrynol. Pol.	43	Adiponectin in serum
Jin et al. [23]	2016	Obstet. Gynecol. Sci.	52	Adiponectin in plasma
Feng et al. [33]	2018	Trop. J. Pharm. Res.	46	Adiponectin in serum
				Adiponectin in ascites
Lane et al. [34]	2011	BMC Cancer	39	IL-6 in ascites
Lane at al. [35]	2015	BMC Cancer	53	IL-6 in ascites
Chudecka-Głaz et al. [36]	2015	Onco. Targets. Ther.	36	IL-6 in ascites
Kampan et al. [37]	2020	Sci. Rep.	18	IL-6 in ascites
			51	IL-6 in serum
Matte et al. [38]	2012	Am. J. Cancer Res.	38	IL-6 in ascites
Canton-Romero et al. [39]	2017	Oxid. Med. Cell. Longev.	21	IL-6 in ascites
Maccio et al. [40]	2009	J. Cell. Mol. Med.	104	IL-6 in plasma
Giuntoli et al. [24]	2009	Anticancer. Res.	37	IL-6 in ascites
			33	IL-6 in serum
Mikuła-Pietrasik et al. [25]	2016	Cell. Oncol.	16	IL-8 in ascites
			8	MCP-1 in ascites
Zhang et al. [26]	2019	Oncol. Lett.	92	IL-8 in serum
Sanguinete et al. [27]	2017	Immunol. Invest.	26	IL-8 in serum
Sadłecki et al. [28]	2011	Ginekol. Pol.	21	IL-8 in ascites
			21	IL-8 in serum
			8	MCP-1 in ascites
Radestad et al. [29]	2019	Oncoimmunology	35	IL-8 in ascites
			35	IL-8 in serum
Hefler et al. [30]	1999	Br. J. Cancer	86	MCP-1 in serum
Schutyser et al. [31]	2002	J. Biol. Chem.	12	MCP-1 in ascites
Mahner et al. [32]	2010	BMC Cancer	37	TIMP-1 in serum

IL-6—interleukin 6, IL-8—interleukin 8, MCP-1—monocyte chemotactic protein-1, TIMP-1—tissue inhibitor of metalloproteinase-1.

**Table 2:** Concentration of adiponectin in blood (plasma/**serum**).

Disease	Concentration [ng/mL]	No. of Cases	Ref.
Ovarian cancer prior to chemotherapy	**8830.00 ± 3190.00**	43	[22]
Ovarian cancer after chemotherapy	**10,370.00 ± 4180.00**		
Ovarian cancer	8250.00 ± 970.00	52	[23]
Control group—no ovary conditions	11,440.00 ± 1130.00	18	
FIGO I-IV ovarian cancer	**range (~3900.00 ± 400.00 to ~4800.00 ± 700.00)**	46	[33]
Control group—no ovary conditions	**~3400.00 ± 300.00**	17	

FIGO I-IV—The International Federation of Gynecology and Obstetrics classification of ovarian tumor. In column 2, the serum concentrations are marked in bold, and the plasma concentrations in normal type.

**Table 3:** Concentration of interleukin 6 (IL-6) in ascites.

Disease	Concentration [pg/mL]	No. of Cases	Ref.
Ovarian cancer	6419.00 ± 1409.00	39	[34]
FIGO III/IV ovarian cancer	1820.00 (range 279.00–4327.00)	53	[35]
Control group—benign gynecological conditions	15.00 (range 6.00–65.00)	10	
Malignant ovarian cancer	379.00 (range 9.70–528.10)	36	[36]
Control group—benign gynecological conditions	137.80 (range 0.08–528.10)	38	
Advanced ovarian cancer	18,050.00 (range 5162.00–122,883.00)	18	[37]
FIGO III/IV ovarian cancer	3089.00 (range 0.00–31,142.00)	26	[38]
FIGO I/II ovarian cancer	455.00 (range 0.00–29,669.00)	12	
Platinum-refractory FIGO III–IV ovarian cancer	1382.30 ± 257.60	7	[39]
Platinum-resistant FIGO III–IV ovarian cancer	969.60 ± 76.30	4	
Platinum-sensitive FIGO I–IV ovarian cancer	1582.00 ± 346.10	10	
FIGO III/IV ovarian cancer	14,845.28 ± 4658.05	37	[24]

FIGO I-IV—The International Federation of Gynecology and Obstetrics classification of ovarian tumors.

**Table 4:** Concentrations of interleukin 6 (IL-6) in blood (plasma/**serum**).

Disease	Concentration [pg/mL]	No. of Cases	Ref.
Advanced ovarian cancer	**28.30**	33	[37]
Ovarian cancer with accompanying ascites	**53.00 (range 11.20–216.00)**	18	
Control group—benign gynecological conditions	**7.40**	12	
Control group—no ovary conditions	**1.20**	21	
FIGO III/IV ovarian cancer	**29.97 ± 5.14**	33	[24]
Platinum-refractory FIGO III–IV ovarian cancer	396.60 ± 105.00	7	[39]
Platinum-resistant FIGO III–IV ovarian cancer	834.20 ± 31.00	4	
Platinum-sensitive FIGO I–IV ovarian cancer	936.40 ± 284.60	10	
Control group—no ovary conditions	448.34 ± 28.00	6	
FIGO III/IV ovarian cancer	18.60 ± 18.70	75	[40]
FIGO I/II ovarian cancer	8.10 ± 10.40	29	
Control group—no ovary conditions	3.00 ± 3.20	95	

FIGO—The International Federation of Gynecology and Obstetrics. In column 2, the serum concentrations are marked in bold, and the plasma concentrations in normal type.

**Table 5:** Concentrations of interleukin 8 (IL-8) in ascites.

Disease	Concentration [pg/mL]	No. of Cases	Ref.
Ovarian cancer	396.60 *	21	[28]
FIGO III and IV ovarian cancer	231.30 (74.70–6669.30) *	35	[29]
FIGO IIIC and IV ovarian cancer	536.00 ± 82.00 ^#^	8	[25]
Benign gynecological conditions	450.00 ± 72.00 ^#^	8	

*—median (range), ^#^—mean ± SEM, FIGO III-IV—The International Federation of Gynecology and Obstetrics classification of ovarian tumors.

**Table 6:** Concentrations of interleukin 8 (IL-8) in blood serum.

Disease	Concentration [pg/mL]	No. of Cases	Ref.
FIGO IIIC ovarian cancer	233.35 (38.60–1222.50) *	5	[27]
FIGO I–IIIB ovarian cancer	95.20 (12.40–1222.50) *	21	
FIGO III–IV ovarian cancer	228.41 ± 6.79	72	[26]
FIGO I–II ovarian cancer	181.70 ± 13.54 ^#^	20	
Benign gynecological conditions	79.68 ± 9.53 ^#^	64	
Control group—no ovary conditions	54.31 ± 10.26 ^#^	58	
Ovarian cancer	22.70 *	21	[28]
FIGO III–IV ovarian cancer	101.70 (10.70–614.20) *	35	[29]

*—median (range), ^#^—mean ± SEM, FIGO—The International Federation of Gynecology and Obstetrics classification of ovarian tumors.

**Table 7:** Concentrations of monocyte chemotactic protein-1 (MCP-1) in ascites.

Disease	Concentration [pg/mL]	No. of Cases	Ref.
FIGO IIIC and IV ovarian cancer	330.00 ± 25.00 *	8	[25]
Benign gynecological conditions	325.00 ± 5.00 *	8	
FIGO IC and IV ovarian cancer	3200.00 ± 400.00 ^$^	12	[31]
Non-gynecological cancers and benign ovary conditions	3300.00 ± 1000.00 ^$^	12	

*—median (range), ^$^—mean value after insulation, FIGO—The International Federation of Gynecology and Obstetrics classification of ovarian tumors.

**Table 8:** Concentrations of monocyte chemotactic protein-1 (MCP-1) in blood serum.

Disease	Concentration [pg/mL]	No. of Cases	Ref.
FIGO I–III ovarian cancer	535.60 (129.60–1200.00) *	48	[30]
Relapsing ovarian cancer	427.30 (193.40–1101.00) *	38	
Benign gynecological conditions	371.20 (222.00–986.80) *	67	
Control group of healthy women	318.70 (241.30–681.40)	45	

*—median (range), FIGO I-III—The International Federation of Gynecology and Obstetrics classification of ovarian tumors.

**Table 9:** Concentrations of tissue inhibitor of metalloproteinase-1 (TIMP-1) in the blood serum of patients with ovarian cancer.

Disease	Concentration [pg/mL]	No. of Cases	Ref.
FIGO I–IV ovarian cancer before cytoreductive procedure	403.00 (273.00–887.00) *	37	[32]
FIGO I–IV ovarian cancer after cytoreductive procedure	529.00 (323.00–1000.00) *		
FIGO I–IV ovarian cancer during chemotherapy	351.00 (204.00–616.00) *		
FIGO I–IV ovarian cancer after chemotherapy	333.00 (209.00–990.00) *		

*—median (range), FIGO I-IV—The International Federation of Gynecology and Obstetrics classification of ovarian tumors.

**Table 10:** Summary of the selected adipokine concentrations in the blood and ascites of patients with ovarian cancer.

Adipokine	Ovarian Cancer	
	Blood	Ascites
Adiponectin	↓ or ↑	N/A
Interleukin 6 (IL-6)	↑	↑↑
Interleukin 8 (IL-8)	↑	↑
Monocyte chemotactic protein-1 (MCP-1)	↑	↑ ^#^
Tissue inhibitor of metalloproteinase-1 (TIMP-1)	↑ *	N/A

*—compared with benign gynecological conditions, ^#^—no statistically significant differences, ↑—increased, ↑↑—strongly increased, ↓—decreased, N/A—not available.
